# Deep groundwater and potential subsurface habitats beneath an Antarctic dry valley

**DOI:** 10.1038/ncomms7831

**Published:** 2015-04-28

**Authors:** J. A. Mikucki, E. Auken, S. Tulaczyk, R. A. Virginia, C. Schamper, K. I. Sørensen, P. T. Doran, H. Dugan, N. Foley

**Affiliations:** 1 Department of Microbiology, University of Tennessee, Knoxville, Tennessee 37996, USA; 2 Department of Geosciences, Aarhus University, Aarhus 8000, Denmark; 3 Department of Earth and Planetary Sciences, University of California, Santa Cruz, California 95064, USA; 4 Environmental Studies Program, Dartmouth College, Hanover, New Hampshire 03755, USA; 5 Sorbonne Universités, UPMC Univ Paris 06, CNRS, EPHE, UMR 7619 Metis, 4 place Jussieu, Paris 75252, France; 6 Department of Geology and Geophysics, Louisiana State University, Baton Rouge, Louisiana 70803, USA; 7 Department of Earth and Environmental Sciences, University of Illinois at Chicago, Chicago, Illinois 60607, USA

## Abstract

The occurrence of groundwater in Antarctica, particularly in the ice-free regions and along the coastal margins is poorly understood. Here we use an airborne transient electromagnetic (AEM) sensor to produce extensive imagery of resistivity beneath Taylor Valley. Regional-scale zones of low subsurface resistivity were detected that are inconsistent with the high resistivity of glacier ice or dry permafrost in this region. We interpret these results as an indication that liquid, with sufficiently high solute content, exists at temperatures well below freezing and considered within the range suitable for microbial life. These inferred brines are widespread within permafrost and extend below glaciers and lakes. One system emanates from below Taylor Glacier into Lake Bonney and a second system connects the ocean with the eastern 18 km of the valley. A connection between these two basins was not detected to the depth limitation of the AEM survey (∼350 m).

## Abstract (web summary)

Conditions below the active permafrost layer in the McMurdo Dry Valleys, Antarctica, are thought to be ice cemented. Here, the authors use an airborne electromagnetic sensor to image the resistivity beneath the valley floor, which indicates the presence of high-salinity liquids at temperatures well below freezing.

## Graphical abstract

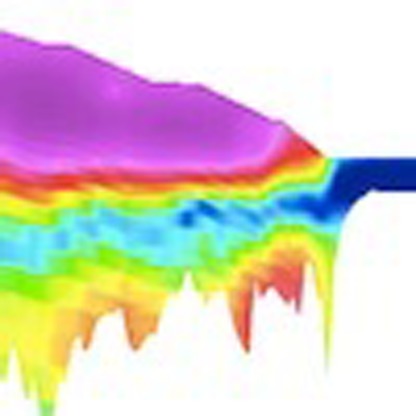

Our understanding of Antarctica's subsurface environments has advanced dramatically in recent decades. We now know that subglacial water is widespread with at least half of the areas covered by the Antarctic ice sheet having aqueous systems beneath that are analogous to lakes and wetlands on other continents^1^,^2^,^3^. However, little is known about groundwater in Antarctica's ice-free regions and connectivity of these fluids to the coastal margins. Recent measurements have shown direct submarine groundwater discharge near Lützow–Holm Bay, Antarctica^4^ and the volumes of these groundwater contributions from the continent to the Southern Ocean may be significant^5^. While very few of these subsurface aquatic environments have been sampled, all have harboured microorganisms^6^,^7^,^8^. The metabolic activity of these microbial communities enhances mineral weathering, resulting in the subsequent release of solutes, see, for example, refs 9, 10 such that subglacial groundwater discharges may contribute a significant flux of essential nutrients to affect near-shore lacustrine and marine productivity^11^,^12^.

The McMurdo Dry Valleys (MDV), situated along the Ross Sea coastline, is the largest ice-free region in Antarctica^13^. Following their discovery by the Robert Scott expedition of the early twentieth century, international researchers have extensively studied the MDV, beginning with the International Geophysical Year programme in 1957 and continuing with the current US long-term ecological research programme. The McMurdo long-term ecological research programme^14^ was established in 1993 and provides the longest continuous record of physical and biological information for Taylor Valley (Fig. 1a) and other locales in the MDV. Our current understanding of hydrological linkages in the MDV is based primarily on observed surface processes^15^. Local glaciers are defined as cold based with beds below the pressure melting temperature of freshwater^16^. On seasonal timescales, supraglacial melt generated during the austral summer feeds perennial streams, which then interact with desert soils dissolving solutes and redistributing nutrients, ultimately transferring nutrients to ice-covered lakes^14^. On longer timescales, the size and chemistry of the ice-covered lakes fluctuate in response to climate^17^. These changing paleolake levels are thought to create ecological resource legacies of salts, organic matter and landscape change that influence contemporary ecosystem production and biodiversity^18^,^19^,^20^.

Permafrost in the MDV is continuous with a thin active layer (<70 cm depth). The upper 1 m is comprised primarily of ice-cemented and dry-frozen ground and contains a smaller fraction of ground or buried ice^21^. Soil warming can generate near-surface groundwater, identified as surface seeps or water tracks, which concentrate solutes from salt-rich soils along the permafrost boundary^22^,^23^,^24^,^25^. Considerably less is known about the occurrence of groundwater below the active layer; however, conditions below 1 m are assumed to be primarily ice cemented. Several boreholes were drilled in this region as part of the Antarctic Dry Valley Drilling Project (DVDP) in the 1970s, which included isolated seismic surveys and several small-scale ground-based resistivity depth soundings^22^,^26^. Investigators from the DVDP hypothesized the presence of a groundwater system in the Taylor and Wright Valley based on a few surveys of seismic velocities and semi-quantitative DC resistivity measurements^22^,^27^. However, borehole data did not strongly corroborate this idea, and suggested, instead, that frozen ground in the MDV was thicker than predicted. Regardless, the limited spatial coverage of a small number of boreholes restricted extrapolation of subsurface features to the greater MDV system^26^.

A unique feature, known as Blood Falls appears at the snout of the Taylor Glacier in the upper Taylor Valley (Fig. 1a). Blood Falls forms as the weight of the glacier pressurizes subglacial materials leading to the expulsion of a cryoconcentrated brine at the glacier front that flows into Lake Bonney. The brine that emanates is ferrous and stains the glacier a deep crimson colour as it oxidizes at the surface^7^. Blood Falls provides striking surface evidence of deep subsurface liquid in the MDV. The brine has been shown to contain a diverse microbial community that is metabolically active and influences weathering and the geochemistry of the subglacial fluid by liberating ions such as iron and silica from subglacial bedrock^7^,^28^.

Electrical resistivity values increase by several orders of magnitude when soil water freezes^29^ allowing resistivities to be used as an indicator of the temperature and moisture content of subsurface materials. Airborne electromagnetic (AEM) systems have been used successfully in temperate environments to map groundwater resources^30^ and saline coastal systems^31^. Recently, AEM has been used in the Arctic to map the extent of permafrost in Alaska^32^. Surveys based on similar, but ground-based, technologies have also been used in the Arctic to delineate taliks within permafrost^33^,^34^,^35^ and to map permafrost and buried ice features on Livingston Island, Antarctica^36^.

The presence of Blood Falls and the implications for a deeper brine ecosystem represented by this feature, motivated the first ever landscape scale survey of subsurface resistivity in Antarctica. Here we used an AEM sensor called SkyTEM^37^, which induces subsurface electromagnetic currents using a high-powered transmitter loop flown above the surface by helicopter to map features in the MDV. We discuss results from our MDV AEM resistivity survey and the possible geologic and climate histories leading to the formation of different subsurface brine systems (derived from ancient marine waters and/or more recent paleolakes). We further consider the implications of these brine networks for a deep biosphere and hydrological and geochemical connectivity between the marine system and continental subglacial environments.

## Results

### Ground truth for the AEM survey of Taylor Valley

**Figure 1:**
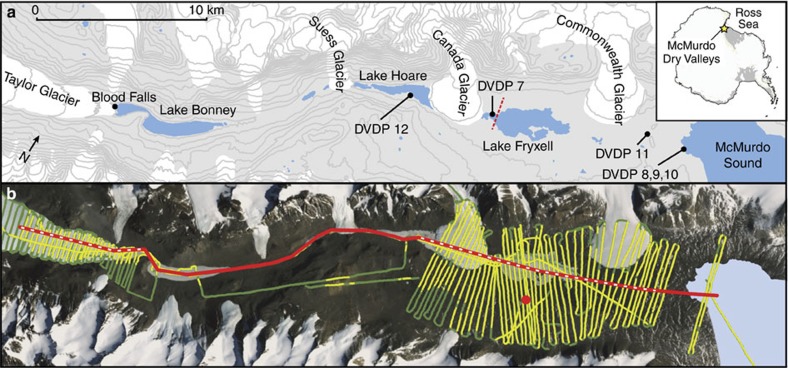
Map of Taylor Valley in Antarctica. (**a**) Map of major lakes, glaciers and DVDP boreholes in Taylor Valley, Antarctica. Dotted red line indicates the location of the Lake Fryxell DVDP geophysical survey (Supplementary Fig. 2) (**b**) AEM flight lines in green with survey lines for which data were processed shown in yellow. Terrain surveyed in this paper is highlighted in red. Dashed line indicates regions where higher-resolution surveys were conducted in the Bonney (Fig. 4) and Fryxell Basins (Fig. 5). Red circle indicates the location of the example SkyTEM sounding (Supplementary Fig. 3).

**Figure 2:**
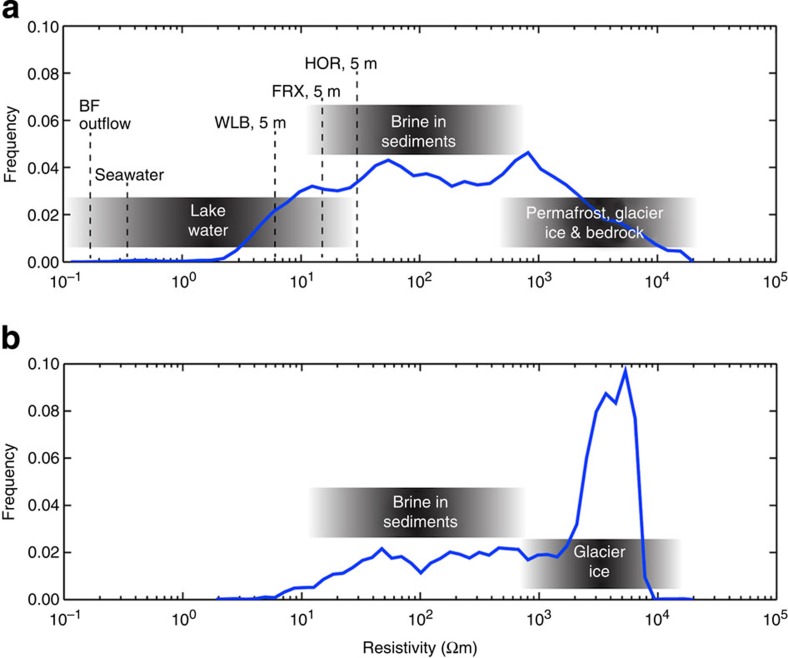
Resistivity histograms from Taylor Valley survey. Estimated resistivity ranges for lake water, brine in sediments and permafrost/glacier ice and bedrock are indicated. The two histograms were derived from the AEM data (**a**) Lower Taylor Valley *in situ* measurements marked: FRX, Lake Fryxell; HOR, Lake Hoare; WLB, West Lake Bonney (depth of measurement in metres follows abbreviation); BF, Blood Falls. (**b**) Histogram of data from Taylor Glacier with measurements from nearby Lake Bonney removed.

We targeted the upper Taylor Valley (Fig. 1a) including lower Taylor Glacier, Blood Falls and the west lobe of Lake Bonney and the lower Taylor Valley from Suess Glacier to the coastline of McMurdo Sound. The AEM approach allowed us to survey the majority of the Taylor Valley, covering ∼295 km^2^ (Fig. 1b). Derived subsurface resistivities spanned four orders of magnitude (Fig. 2) and binned into diverse, but distinct populations that we used to classify landscape units observed more broadly in the MDV. These represent approximate resistivity ranges for lake water (∼0.1–30 Ωm), partially unfrozen, brine-containing sediments (∼10–800 Ωm) and permafrost/glacier ice (∼500–20,000 Ωm). These landscape unit classifications are based on our own observations, comparisons with earlier work in the MDV, see, for example, ref. 27 (Table 1), similar surveys in the Arctic^33^ and accepted electromagnetic interpretations^38^.

### Evidence for extensive subsurface brine systems

**Figure 3:**
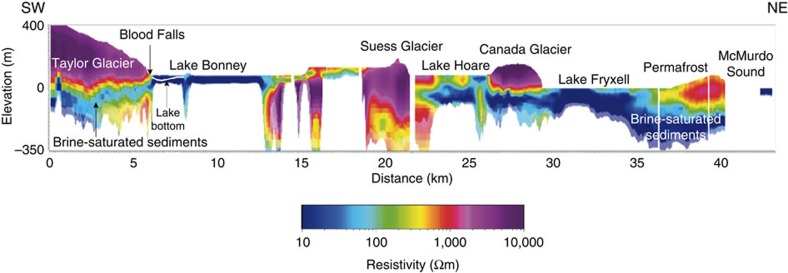
Resistivity cross-section for the length of the Taylor Valley. Resistivity profile along the length of the Taylor Valley (flight line denoted in red in Fig. 1b). Low resistivities near McMurdo Sound to Lake Hoare interpreted as hydrological connectivity of brine in sediments extending from the coastal margin inland and beneath the Canada Glacier. To the west, resistivities increase below Suess Glacier and again towards Lake Bonney. In the Bonney Basin, low-resistivity patterns suggest connectivity of brine-rich sediments below the Taylor Glacier with proglacial Lake Bonney at the glacier terminus and near the location of Blood Falls.

We detected two distinct zones of low resistivity in Taylor Valley (Fig. 3). One occurs in the upper Taylor Valley extending from below Taylor Glacier into Lake Bonney (Fig. 3, 0–13 km). A separate deep (>100 m) low-resistivity zone is located below the Suess Glacier, ∼18 km inland, extending from Lake Hoare under the Canada Glacier and into Lake Fryxell, ultimately connecting to McMurdo Sound (Fig. 3, 18–40 km).

### The Taylor Glacier and Bonney Basin

**Figure 4:**
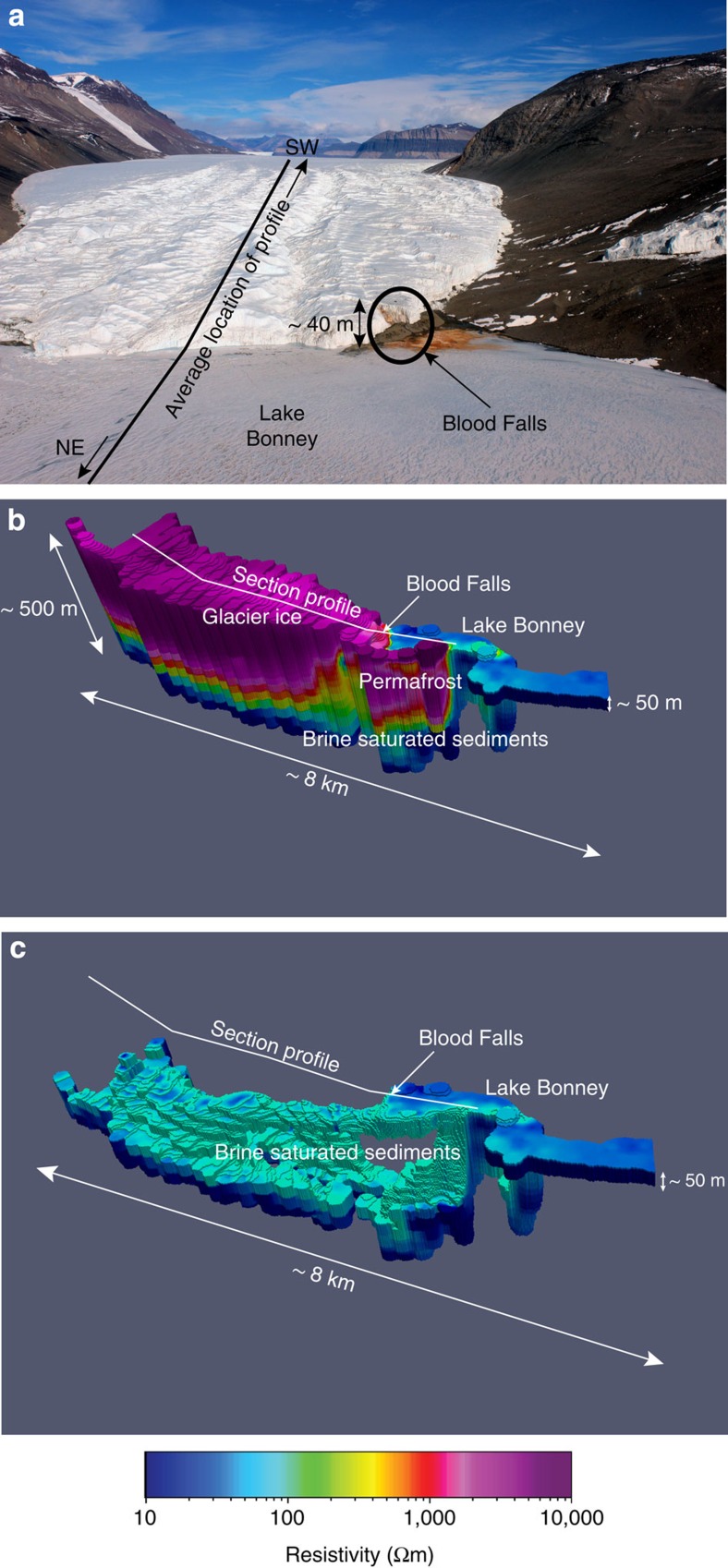
Resistivity maps from the Taylor Glacier and Bonney Basin survey. (**a**) Photograph of the lower 2 km of Taylor Glacier showing its contact with Lake Bonney. Blood Falls is marked by the orange staining of ice caused by the release and oxidation of subglacial brine. (**b**) 3D presentation showing highly resistive glacier ice and permafrost with conductive subglacial brine. (**c**) 3D image of the Taylor Glacier with resistivities above 100 Ωm removed to show the spatial extent of conductive brine-saturated sediments below glacial ice.

AEM exploration of the lower Taylor Glacier revealed the presence of an extensive low-resistivity zone that we interpret to represent a brine system that connects subglacial fluid with Lake Bonney (Fig. 3). The sensor was flown over the lower 5 km of the Taylor Glacier (Fig. 4) and successfully penetrated up to 350 m of ice. Beyond about 6 km up-glacier from the terminus, the ice was too thick to obtain a reliable signal. Highly resistive glacier ice overlies low-resistivity brine in sediments for the length of the glacier surveyed (Fig. 4b,c).

### Lower Taylor Valley and Fryxell Basin

**Figure 5:**
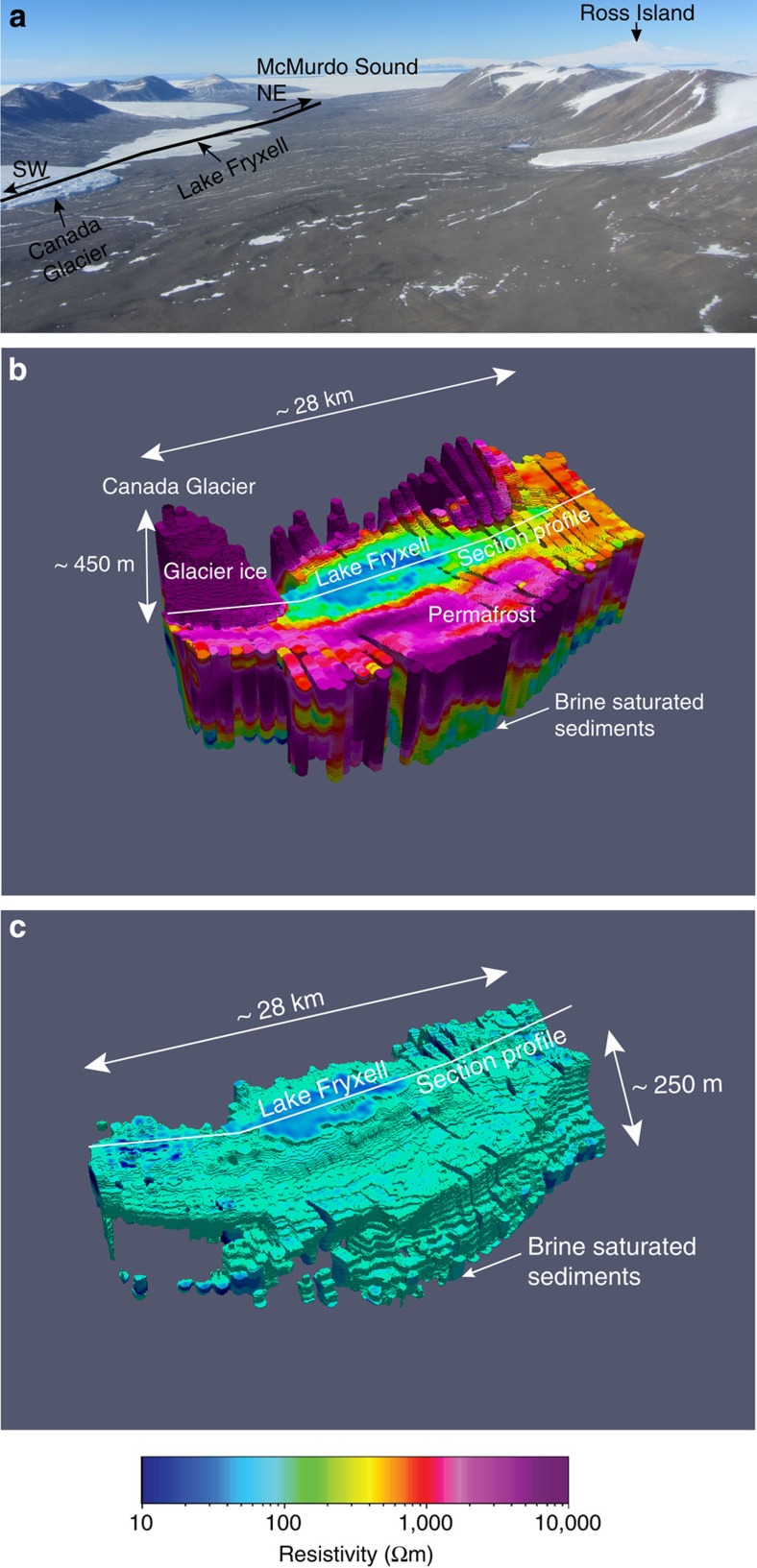
Resistivity maps from the Lower Taylor Valley survey. (**a**) Photograph of the Lake Fryxell Basin survey area with the profile line indicated in black. McMurdo Sound is to the east (**b**) 3D presentation of resistive glacier ice and permafrost with conductive lake water and brine-saturated sediments. (**c**) Lake Fryxell Basin image from **b** with resistivities above 100 Ωm removed to show the spatial extent of conductive brine-saturated sediments.

Deep (>100 m) low-resistivity zones that we interpret to represent brine-bearing materials were detected throughout the lower Taylor Valley subsurface with apparent hydrological connection to McMurdo Sound and the Ross Sea (Figs 3a and 5). Widespread low-resistivity layers (10–100 s Ωm), interpreted as evidence for unfrozen material, were detected through the subglacial zone of Canada Glacier and around and below Lake Fryxell (Fig. 5b,c).

## Discussion

Our AEM survey produced regional-scale resistivity data that confirms and expands the overall extent of permafrost and reveals two extensive subsurface brine systems in the MDV. The AEM data correlate well with conductivity profiles from the MDV lakes (Table 1). For example, the hypersaline bottom waters of the west lobe of Lake Bonney (30–35 m depth) have a resistivity of 0.13–0.12 Ωm, and the AEM returned a resistivity value of 0.42 Ωm for this depth interval. The AEM sensor was flown over the site of the DVDP ground-based resistivity survey and boreholes in the Fryxell Basin (Fig. 1) and consistently recorded resistivity around 100 Ωm or less at depths below 185 m, indicating the presence of brine in sediments. Although not directly comparable, our results are consistent with previous DVDP geophysical measurements^26^ (Table 1; Supplementary Fig. 2). For example, when DVDP Borehole 10 (Fig. 1a; near the McMurdo Sound) penetrated the permafrost layer at 183 m below sea level (b.s.l.), liquid entered the borehole rising to ∼125 m (ref. 26). This borehole fluid was twice the salinity of seawater with an *in situ* temperature of −4 °C (ref. 26). Further inland at DVDP 11, drilling fluid drained from the borehole at ∼248 m b.s.l. The loss of drilling fluid suggested that the drill penetrated the confining layer (described by Cartwright and Harris^26^ as ‘the interface between frozen ground and liquid groundwater') before entering an aquifer. Temperature measurements collected from boreholes (DVDP 10–12) in the Taylor Valley were above −10 °C at depths greater than 100 m from the surface^39^. Given the salinities of the sediments at these depths^40^, porewater would remain liquid. Collectively these earlier observations and the low-resistivity values detected with AEM (<100 Ωm) support our interpretation of the presence of two distinct brine groundwater systems in the Taylor Valley.

Taylor Glacier is a well-studied polar outlet glacier representative of ice drainage pathways in the cold margins of the Antarctic ice sheet, see, for example, ref. 16. Thermodynamic models of ice temperature^41^ distribution in Taylor Glacier indicates that basal temperatures are well below the pressure melting point of ice and therefore it had been considered predominantly cold based, moving through internal ice deformation. The area of lowest resistivity below Taylor Glacier corresponded with a topographic overdeepening of 80 m b.s.l. at ∼5.75 km up-glacier from the terminus at Lake Bonney (Fig. 3). Hubbard *et al*.^41^ measured high bed reflectance at this same location with ice-penetrating radar; both this radar data and our AEM measurements are indicative of subglacial hypersaline liquid. The radar survey, however, did not detect evidence of saturated sediments at the glacier snout in the vicinity of Blood Falls nor did it reveal the deep connectivity with Lake Bonney that AEM was able to resolve (Figs 3 and 4c). AEM confirms the presence of unfrozen water at the base of Taylor Glacier (Figs 3 and 4), likely because its freezing point is depressed by salts, and to a much smaller degree, pressure from the overlying ice. From the AEM data, we estimate a volume of 1.5 km^3^ for subglacial brine-saturated sediments below Taylor Glacier (Fig. 4). Porosity in glacial sediments varies but reported values are typically in the range of 20% or higher^42^. The ANDRILL AND-2A core drilled near the mouth of Taylor Valley yielded ∼20–30% porosity in Late Quaternary glacigenic sediments^43^. In the saturated sediments below Taylor Glacier, a porosity of just 12% is required for a subglacial brine volume equivalent to the water column volumes of Lakes Bonney, Fryxell and Hoare combined (∼0.18 km^3^).

Geologic evidence indicates that the MDV was a fjord ecosystem during the Miocene when seawater intruded Taylor Valley beyond the current extent of the Taylor Glacier^44^,^45^. Subsequent climatic cooling may have led to a build-up of salts through freezing (cryoconcentation) of the saline water^46^ creating dense brines. Our data indicate that this brine still exists beneath the Taylor Glacier (Figs 3 and 4) an inference that is further supported by the presence of Blood Falls (Fig. 4a). Low-resistivity (<0.17 Ωm) subglacial water discharges from Blood Falls intermittently and is saline enough to remain liquid to temperatures as low as −6 °C at atmospheric pressure^7^. Multiple lines of evidence support a marine origin of this subglacial effluent: the major ions were present in marine ratios (Na:Cl=0.88 in Blood Falls; Seawater=0.86)^46^, the δ^37^Cl signature was marine (∼0.0‰)^46^ and genomic material and bacterial isolates recovered from the brine were phylogenetically related to marine lineages^47^. Our AEM results suggest that discharges at Blood Falls are sourced from a more regionally extensive body of subglacial brine and not a small-scale feature confined to the terminus of Taylor Glacier. Such cryogenically concentrated fluids may underlie other parts of the Antarctic ice sheet margins. Findings presented here suggest that other parts of the ice sheets with beds below the pressure melting point of freshwater ice may contain liquid water and may move through basal sliding^48^, rather than internal deformation alone.

The unfrozen brines under the surveyed lakes (Figs 3 and 5) could be accounted for by solute concentration due to freezing and/or evaporation events of a large paleolake, see, for example, ref. 49. Models based on radiocarbon chronology of perched deltas, shorelines and other lake deposits suggest that Glacial Lake Washburn occupied much of Taylor Valley during the Last Glacial Maximum up to an elevation of ∼300 m above sea level (a.s.l.)^17^,^50^,^51^. However, soluble salt accumulation in MDV soils suggests that Lake Washburn only occupied the west end of the valley up to the same elevation^49^. Following retreat of the Ross Sea Ice Sheet, smaller lakes occupied Taylor Valley in both ends up to ∼120 m a.s.l. as controlled by lake sills or spill points. Geochemical profiles in the current water columns^46^ indicate that, within the past 1,000 years, lake levels in the Taylor Valley were lower than present day. Thus, the current lakes appear to be remnants of these larger paleolakes following periods of major drawdowns to small ponds or even complete evaporation, with subsequent refilling with less saline waters to modern day levels^52^,^53^. As lakes in the Taylor Valley lowered and concentrated, dense bottom brine would have infiltrated the highly permeable glacial till in the basin, sinking within the subsurface, similar to the above proposed formation of the brine below Taylor Glacier. Alternatively, these subsurface brines could be a legacy of much older marine deposition. The presence of unfrozen soil extending beyond the current lake margins to elevations approximating the estimates of a high stand Glacial Lake Washburn (Fig. 5) supports the large lake hypothesis of Hall and Denton^51^.

**Figure 6:**
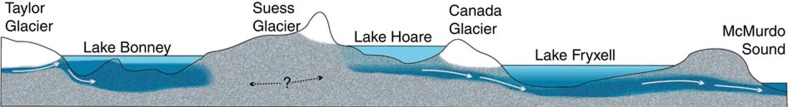
Conceptual diagram depicting predicted hydrological connectivity. Two distinct regions of subsurface brine were identified in the MDV. The ‘?' indicates the zone between Lake Bonney and Lake Hoare where no connectivity was identified with our survey.

Previous to our study, the MDV lakes were viewed as being isolated from one another. From the surface, Canada Glacier appears to be preventing communication between the surface waters of Lakes Hoare and Fryxell (Fig. 1a). However, our data suggest that there is flow from the bottom of Lake Hoare into Lake Fryxell (Fig. 3 and Fig. 6). The implication of this to the geochemistry of the lakes is profound. It was previously thought that Lake Hoare completely evaporated around 1,200 years ago and its salts blew away. In this model, the relatively fresh, modern Lake Hoare resulted from a subsequent refilling with Canada Glacier melt waters^52^. An alternative hypothesis for dilute Lake Hoare water is that Lake Hoare is a headwater lake in our groundwater system. Lake Fryxell on the other hand is more brackish as it is receiving some portion of its bottom water from the groundwater flow system. Lake Bonney has the most saline bottom water in the valleys, which similarly may be related to its position as a terminal lake in a separate groundwater system receiving contributions from the saline subglacial marine brines from beneath Taylor Glacier (Fig. 3).

The weight of Canada Glacier could cause subsurface discharge at the glacier terminus and/or into Lakes Hoare and Fryxell. Our AEM data indicate that Canada Glacier has over-ridden what we interpret as lake water and brine-saturated sediments however a surface discharge feature has not formed. Discharge sourced from beneath Canada Glacier would involve squeezing of local groundwater or recycling of proglacial lake water. Thus, the lack of a Blood Falls like feature at Canada Glacier supports the model for Taylor Glacier and Blood Falls, where discharge requires an upstream brine supply.

Blood Falls is the only known surface manifestation of these deep brine systems and has been shown to contain a viable ecosystem with numerable microbial cells (∼6 × 10^4^ ml^−1^). These numbers are typical for groundwater (∼1 × 10^3^–1 × 10^4^ cells ml^−1^)^54^ and other subglacial environments (∼1 × 10^4^–1 × 10^7^ cells ml^−1^)^2^. Previous work has shown that some of the energy needed to support cellular biosynthesis in this microbial community is gained from oxidation–reduction reactions that involve iron and sulfur, resulting in the liberation of iron as Fe (II)^7^. Silica concentrations in Blood Falls effluent are also high relative to other streams in the MDV^55^, suggesting a high degree of weathering below Taylor Glacier, which is likely enhanced by microorganisms^10^,^28^. If Blood Falls brine is representative of the subsurface fluid observed with AEM, an extensive ecosystem exists below the Taylor Glacier and much of Taylor Valley (Figs 3 and 6). DVDP borehole temperature logs indicate that *in situ* temperatures at depths where resistivity is indicative of liquid range between −3 to −20 °C (ref. 39), temperatures considered within the range suitable for microbial life^56^. Thus, the relative frequency of resistivity measurements across the Lower Taylor Valley (Fig. 2) shows the prevalence of potential habitats where temperature, salinity and liquid water might combine to support life.

Brine systems within and below permafrost along Antarctica's coastal margins may influence surface ecosystem processes. Blood Falls reveals how microbial metabolism can release iron from underlying bedrock, which is ultimately discharged to the surface or below ground to Lake Bonney. Two major contributions of bioavailable iron to the Southern Ocean include aeolian dust (0.01–0.13 Tg per year) and nanoparticulate iron (0.06–0.12 Tg per year) in iceberg entrained sediments^57^. Submarine groundwater discharge, is another unaccounted for, and potentially vital source of iron and silica to a micronutrient limited Southern Ocean^11^. Release events at Blood Falls are episodic. We calculate, based on a surface discharge estimate of ∼2,000 m^3^ in volume^58^ with Fe and Si concentrations in outflow of 3.2 mM (ref. 7) and 264 μM (ref. 55), respectively, that a release event can deliver ∼420 kg of bioavailable Fe and 13.5 kg of Si to proglacial Lake Bonney. While similar subglacial outflow events of coastal glaciers might represent small, episodic releases of growth-limiting micronutrients, these pulses could still significantly enhance lake or near-shore marine productivity. Discharge events like at Blood Falls would represent only a small fraction of the subsurface groundwater discharge possible along coastal margins. The total flux of these nutrients remains poorly resolved; however, a recent report estimates iron flux from ice sheet meltwaters at 0.06–0.17 Tg per year, which is comparable to aeolian fluxes to polar waters^59^. If Antarctic submarine groundwater discharge is relatively rich in dissolved iron, for instance, if it has the concentration of iron comparable to that in Blood Falls brines, then it would only take a modest discharge of approximately 0.3–0.9 km^3^ to supply 0.06–0.17 Tg per year of Fe to the Southern Ocean. This represents about 0.5–1.5% of the total annual subglacial meltwater production estimated for Antarctica (∼60 km^3^)^60^. On other continents, submarine groundwater discharge represents a much higher fraction of their total surface water inputs, 6–10% (ref. 61). The paucity of constraints on groundwater pressure gradients and hydraulic conductivity distribution in Taylor Valley prohibits us from estimating the specific regional contribution of submarine groundwater discharge.

The subpermafrost brines in the MDV provide an important terrestrial analogue for future exploration of a subsurface Martian habitat. Briny groundwater has been suggested as supporting a deep biosphere on Mars^62^. Recent mineralogical analysis of Gale Crater supports the notion that previous fluvio-lacustrine environments may have hosted chemoautotrophic microorganisms^63^. On Mars, as we observe in the dry valleys, connectivity between lacustrine systems and groundwater would be important in sustaining ecosystems through drastic climate change, such as lake dry-down events^63^.

On the basis of the first AEM study of the MDV region, we conclude that a deep briny groundwater system exists beneath glaciers, lakes and permafrost in Taylor Valley (Fig. 6). These brines appear related to the long-term geological history of the MDV and may represent ancient changes in sea level and subsequent marine intrusion and the draw-down of paleolakes linked to the Last Glacial Maximum and recent climate variation. We observed geophysical evidence of hydrological connectivity between lakes, which were previously assumed to be isolated from one another. This finding has significant implications for interpreting past geochemical models of the evolution of dry valley lake chemistry and biology. The subsurface deep brines contain an active microbial community, as evidenced by the surface release of brine at Blood Falls, Taylor Glacier. Our results also suggest that brine flows towards the coast from ∼18 km inland where it must become submarine discharge. Microbial weathering of mineral substrates in subsurface groundwater discharge may be a significant source of solutes to the Southern Ocean. The subpermafrost brines in the MDV may provide an important terrestrial analogue for future exploration of a subsurface Martian habitat.

## Methods

### The sensor system

SkyTEM is a time-domain electromagnetic, helicopter-borne sensor system (AEM) designed for hydrogeophysical and environmental investigations^29^,^64^. AEM induces eddy currents in the subsurface and measures the decaying (secondary) magnetic fields in a pick-up coil mounted in the tail (Supplementary Fig. 1). The decay rates allow for the distinction between electrically conductive brine-bearing sediments and resistive ice-bearing formations. Electromagnetic (EM) data were acquired using the SkyTEM504 AEM system during the Antarctic austral summer (November–December 2011). Flight lines were ∼200–400 m apart with soundings at 15 m intervals resulting in 1,000 km of acquired flight lines.

The system uses a 500-m^2^ transmitter loop with four wire turns and a maximum current of 95 Amps to induce eddy currents in the subsurface (Supplementary Fig. 2). The physics of a transient system is that a large current in the transmitter coils is turned off abruptly (in a few micro seconds). According to Faraday's Law of induction, this action causes the (primary) magnetic fields to change inducing eddy (secondary) currents in the ground. The decaying magnetic fields from these currents are measured in a pick-up coil mounted in the tail. The rate of change of the currents is related to the subsurface resistivity such that low-resistivity layers such as brines or clays have a slow decay rate and therefore a high secondary magnetic field, while high-resistivity layers such as sand, gravels or permafrost have a high decay rate, hence a low secondary magnetic field. For an in-depth discussion of the physics of AEM systems, we refer to Christiansen *et al*.^65^

### AEM data processing

The continuous data set was acquired at an average flight speed of 55 km h^−1^ and a nominal flight height above the ground of 35 m. The system transmits two magnetic moments (number of turns multiplied by the area multiplied by the current in the transmitter loop), a low moment for resolution of the near-surface layers and a high moment for resolution of the deeper layers (Supplementary Fig. 3a). The sensor in this study was equipped with altimeters and inclinometers to record frame altitude, pitch and roll throughout the survey to correct for deviations from horizontal during flight. Before shipment to Antarctica, the system was calibrated at the Danish national reference site^66^ to ensure correct data levels.

Data (transients and navigation data) were processed in the software package Aarhus Workbench, a software platform for processing and inversion of AEM data^67^. Retrieval of subsurface resistivities is done in a process called inversion where a cost function consisting of the difference between measured and model data and with model regularization constraints minimized in a least square^68^. To achieve maximum credibility of the models resulting from the inversion, raw data was corrected for pitch and roll of the transmitter and receiver loops. Data were filtered at late times to achieve a depth of investigation (DOI) of up to ∼350 m depth without compromising lateral resolution. Flight height is included as an inversion parameter in the inversion scheme with a prior value and a s.d. as determined from the laser altimeters attached to the transmitter frame. The final inversion of the SkyTEM data was done using the quasi three-dimensional (3D) spatially constrained inversion scheme^69^ with models discretizing the subsurface into 30 layers logarithmically distributed from the surface to a depth of about 400 m (Supplementary Fig. 3b). For details of the system and inversion, a technical data report is publically available^68^.

Processing and inversion of the data sets were challenging thus much of the automatic filtering was either turned off or used to guide manual filtering. This was due to the relatively abrupt changes from high signal over the hypersaline lakes to a very weak signal occurring late in the time decays over permafrost soils. Fine-tuning of the algorithm inversion was required so that decays in resistivity over short spatial distances could be observed. The DOI was calculated for each sounding^70^ and is an important measure for accessing how deep the sounding can ‘visualize.' The DOI over low-resistivity lakes is ∼100 m; the DOI over the highly resistive permafrost areas was up to 350 m.

### AEM model projections

Following data processing and inversion, resulting models were gridded in two-dimensional maps, and presented in cross sections (Fig. 3) or in 3D (Figs 4 and 5). A combination of Aarhus Workbench and the 3D visualization software ParaView was used to generate these figures.

## Author contributions

J.A.M., S.T., R.A.V. and E.A. designed the project; J.A.M., E.A., K.I.S. and R.A.V. conducted the field survey; C.S., E.A. processed the SkyTEM data; and all authors contributed to data interpretation and writing the manuscript.

## Additional information

**How to cite this article:** Mikucki, J. A. *et al*. Deep groundwater and potential subsurface habitats beneath an Antarctic dry valley. *Nat. Commun.* 6:6831 doi: 10.1038/ncomms7831 (2015).

## Supplementary Material

Supplementary Information Supplementary Figures 1-3.

## Figures and Tables

**Table 1:** Comparison of AEM resistivity values from this study with selected ground-based measurements.

**Sample**	**AEM (Ωm)**	**Comparison resistivity (Ωm)**	**Method**	**Comments**
Ocean 13–35 m	0.57	0.35	Equation of state at −1.8 °C and 35 PSU	
Liquid distilled water (24 °C)	NA	2.3 × 10^3^	Laboratory DC resistivity^27^	
Frozen distilled water (−24 °C)	NA	1.5 × 10^6^	Laboratory DC resistivity^27^	
				
*MDV lake water columns*				
WLB 4–8.3 m	73.6	9.3–3.5	Conductivity probe Nov 2011	Fresh surface water below the ice cover
WLB 13–18 m	4.25	1.1–0.16	Conductivity probe Nov 2011	Chemocline transition
WLB 35.6–42.4 m	0.21	0.13–0.12	Conductivity probe Nov 2011	Hypersaline bottom waters
FRX 4–8.3 m	37.9	25–3.5	Conductivity probe Nov 2011	Fresh surface water just below the ice cover
FRX 8.3–13 m	8.5	3.4–1. 6	Conductivity probe Nov 2011	Chemocline transition
FRX 13–18 m	1.3	1.6–1.2	Conductivity probe Nov 2011	Brackish bottom waters
				
*Lower Taylor Valley*				
>40 m a.s.l.	∼2,630	25,000	DC resistivity^27^	Fryxell Basin permafrost
40 m b.s.l.	<170	<200	DC resistivity^27^	Fryxell Basin brine-containing sediments
140 m b.s.l.	35	NA		Fryxell Basin brine-containing sediments

AEM, airborne transient electromagnetic sensor; a.s.l., above sea level; b.s.l., below sea level; FRX, Lake Fryxell; MDV, McMurdo Dry Valley; NA, not available; Nov, November; WLB, West Lake Bonney.

Lake water column AEM was compared with water column conductivity probe measurements (converted to resistivity). The conductivity probe is deployed at the deepest portion of each lake as part of the routine monitoring program (data available from www.mcmlter.org). Lower Taylor Valley data is from a ground-based DC resistivity survey using a Wenner and Schlumberger electrode configuration^27^.
