# Supplementary material for: Deep groundwater and potential subsurface habitats beneath an Antarctic dry valley
Source: Nat Commun. 2015 Apr 28;6:6831. doi: 10.1038/ncomms7831 (PMC4423215; doi:10.1038/ncomms7831)
Supplement: Supplementary Information — Supplementary Figures 1-3. [file ncomms7831-s1.pdf]

Supplementary figures: *Mikucki et al.* Deep groundwater and potential subsurface habitats beneath an Antarctic Dry Valley

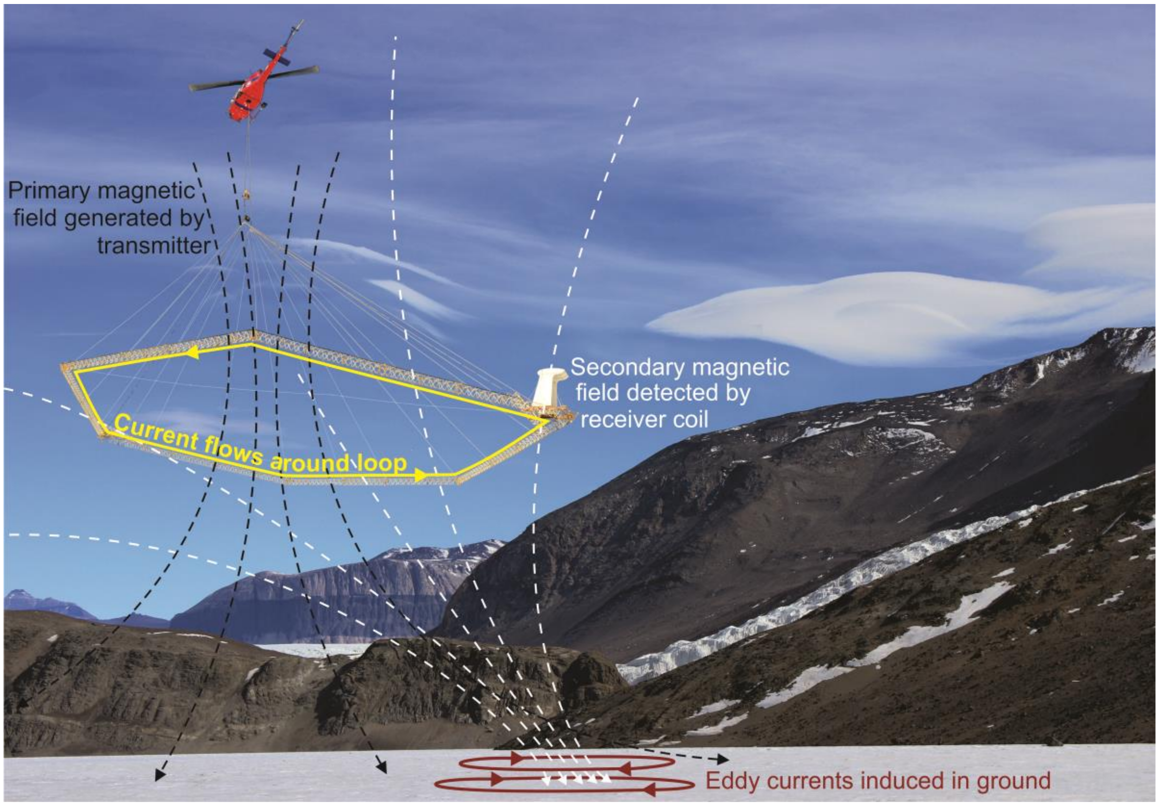

Supplementary Figure 1: A rendition of the SkyTEM system in operation above Lake Bonney. The box between the helicopter and the transmitter lattice contains computers and power supply; the transmitter frame is a constructed wooden lattice. The receiver coil is located at the top of the frame's tail. Cables attached to the frame are transmitter and communication cables. Laser altimeters and angle measurement devices are mounted on the frame. Primary magnetic fields, eddy currents induced in the ground and secondary magnetic fields are drawn to illustrate the physical principles governing the TEM method.

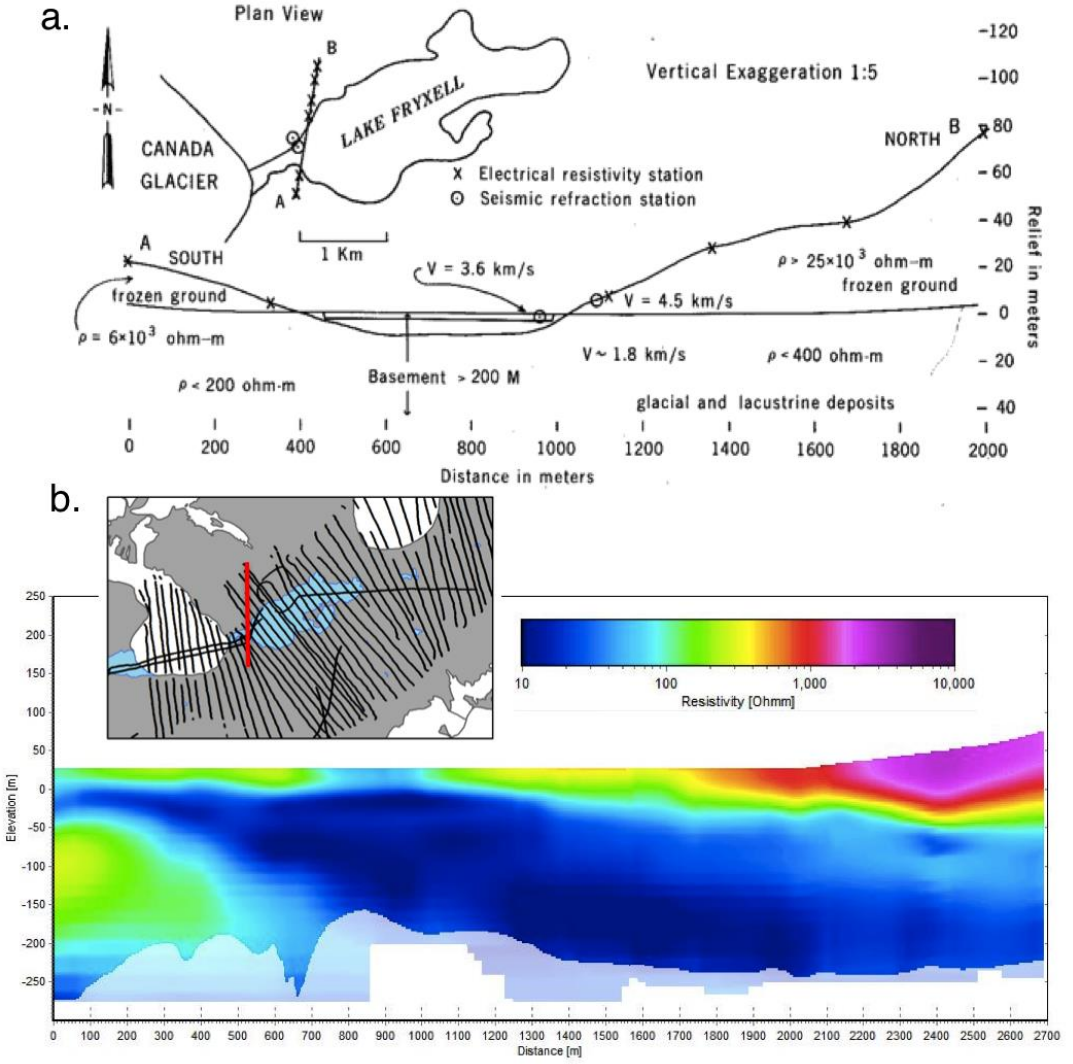

Supplementary Figure 2: Comparison of resistivity surveys in the Lower Taylor Valley. a) Results of a ground-based geophysical survey conducted prior to the DVDP drilling effort (1971-72). Figure adapted from DVDP Bulletin No. 1; available through the Antarctic Marine Geology Research Facility (http://arf.fsu.edu/dry-valley-drilling-project/). These data were generated using a reversed seismic refraction techniques combined with Schlumberger and Wenner electrical resistivity methods (McGinnis et al. 1973). The A-B survey line interpolates soundings from six individual resistivity stations located beside Lake Fryxell. Results show a zone of low resistivity (<400 ohm-m) in the sediments beneath the lake, in comparison to generally high resistivities (> $6x10^3$ ohm-m) in the shallow sediments beside the lake. b) Inset map details AEM coverage and interpolated survey line, similar to that shown above. AEM profile reveals high resistivity permafrost is consistently detected over low resistivity brine in sediments, as predicted by McGinnis et al (1973).

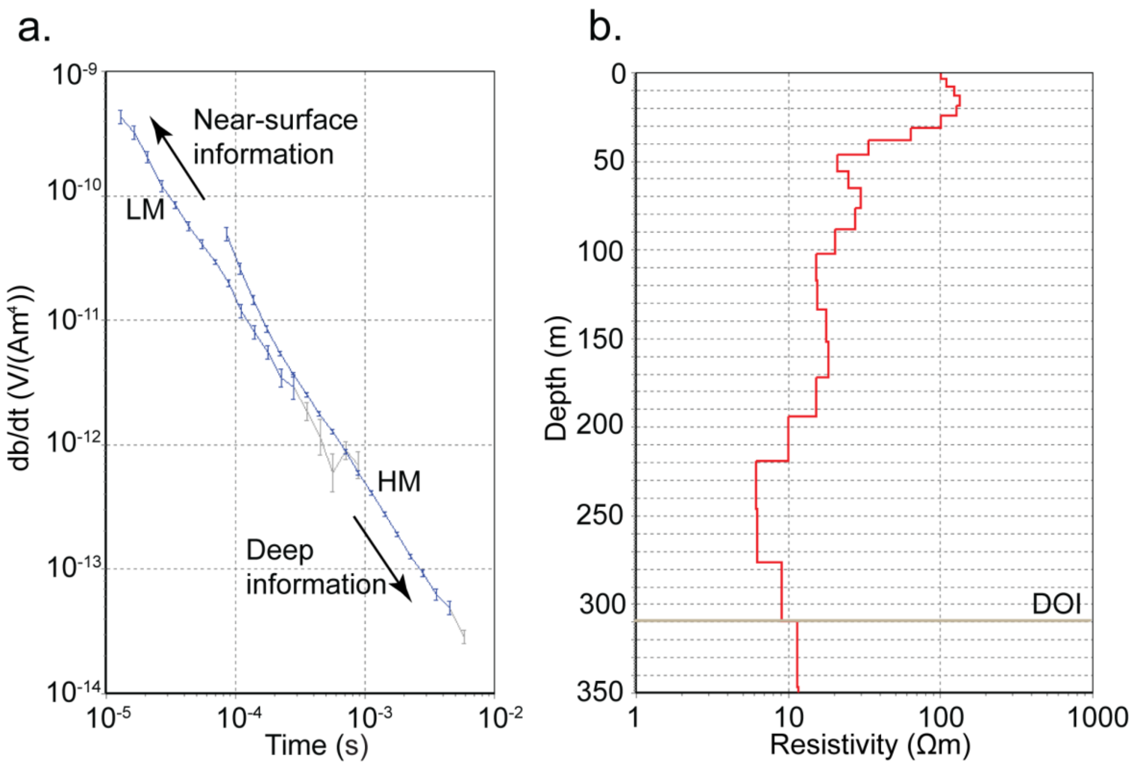

Supplementary Figure 3: Example of a SkyTEM sounding. a) Voltage in the receiver coil measured as the time derivative of the normalized magnetic flux through the coil, db/dt, as a function of time delay after the turn-off of the current. The two moments (LM for low and HM for high) are acquired in order to get both near-surface and deep information Grey data points are noisy and not considered for the inversion; b) corresponding multi-layered model obtained after the inversion, the DOI indicates the estimated depth of investigation.
